# Structure-Function Modeling of Optical Coherence Tomography and Standard Automated Perimetry in the Retina of Patients with Autosomal Dominant Retinitis Pigmentosa

**DOI:** 10.1371/journal.pone.0148022

**Published:** 2016-02-04

**Authors:** Travis B. Smith, Maria Parker, Peter N. Steinkamp, Richard G. Weleber, Ning Smith, David J. Wilson, VPA Clinical Trial Study Group, EZ Working Group

**Affiliations:** 1 Casey Eye Institute, Oregon Health & Science University, Portland, Oregon, United States of America; 2 Center for Health Research, Kaiser Permanente Northwest, Portland, Oregon, United States of America; University of Florida, UNITED STATES

## Abstract

**Purpose:**

To assess relationships between structural and functional biomarkers, including new topographic measures of visual field sensitivity, in patients with autosomal dominant retinitis pigmentosa.

**Methods:**

Spectral domain optical coherence tomography line scans and hill of vision (HOV) sensitivity surfaces from full-field standard automated perimetry were semi-automatically aligned for 60 eyes of 35 patients. Structural biomarkers were extracted from outer retina b-scans along horizontal and vertical midlines. Functional biomarkers were extracted from local sensitivity profiles along the b-scans and from the full visual field. These included topographic measures of functional transition such as the contour of most rapid sensitivity decline around the HOV, herein called HOV slope for convenience. Biomarker relationships were assessed pairwise by coefficients of determination (R^2^) from mixed-effects analysis with automatic model selection.

**Results:**

Structure-function relationships were accurately modeled (conditional R^2^>0.8 in most cases). The best-fit relationship models and correlation patterns for horizontally oriented biomarkers were different than vertically oriented ones. The structural biomarker with the largest number of significant functional correlates was the ellipsoid zone (EZ) width, followed by the total photoreceptor layer thickness. The strongest correlation observed was between EZ width and HOV slope distance (marginal R^2^ = 0.85, p<10^−10^). The mean sensitivity defect at the EZ edge was 7.6 dB. Among all functional biomarkers, the HOV slope mean value, HOV slope mean distance, and maximum sensitivity along the b-scan had the largest number of significant structural correlates.

**Conclusions:**

Topographic slope metrics show promise as functional biomarkers relevant to the transition zone. EZ width is strongly associated with the location of most rapid HOV decline.

## Introduction

Retinitis pigmentosa (RP) is a heterogeneous group of genetic disorders that primarily affects photoreceptors and the retinal pigment epithelium, leading to progressive outer retinal thinning and loss of visual function [1–5]. Understanding the relationships between retinal structure and visual function will lead to improvements in the clinical assessment of RP and optimized endpoint selection for clinical trials. Previous studies of RP patients have described coincident relationships between visual function and several structural biomarkers. These include the thicknesses of the outer nuclear layer [6,7], outer segment [7,8], and outer retina [8,9], and the intactness of the external limiting membrane [9] and the width of the ellipsoid zone (EZ, or the inner segment-outer segment junction) [7,9,10]. The size of the hyperautofluorescent ring has also been correlated with visual function [11]. In particular, a decline in the integrity or extent of the EZ has been associated with RP progression and visual field loss [7,9,12–15]. In these studies, retinal structures were measured with optical coherence tomography (OCT) or fundus autofluorescence imaging and visual function was assessed by multi-focal electroretinography or static perimetry of the central macula.

Our goal was to better characterize the functional correlates of anatomic biomarkers in the retinas of autosomal dominant RP patients. We analyzed a diverse set of biomarkers obtained from full-field standard automated perimetry (SAP) and OCT imaging of clinical trial RP patients. Visual fields were interpolated to facilitate alignment with anatomic imagery and topographic analysis of the visual sensitivity surface. Biomarker relationships were represented by one of three possible candidate models and selected by a statistical quality metric. Biomarker extraction and quantitative modeling were implemented with automated methods wherever possible to maintain objectivity and reproducibility.

## Methods

### Subjects

The subjects were participants in the ongoing Trial of Oral Valproic Acid (VPA) for Retinitis Pigmentosa (NCT01233609). The VPA trial is a phase II multicenter interventional study of the safety and efficacy of valproic acid in a cohort of clinically and genetically confirmed autosomal dominant RP patients. The relevant inclusion criteria for the trial were 20/200 or better visual acuity and 18 years minimum age; exclusion criteria were other retinal diseases present, an intact visual field of less than 5°, and unreliable perimetry measurements in both eyes as determined by the reading center. Patients provided written informed consent for data collection and analysis in accordance with the tenets of the Declaration of Helsinki and an institutional review board (IRB) protocol at each site. All patient records were anonymized and de-identified at the time of collection and prior to analysis. This analysis was determined to be exempt from review by the Oregon Health & Science University IRB in accordance with the Department of Health & Human Services regulation 45 CFR 46.101(b)(4).

To minimize learning effects and preclude any influence from the intervention, our analysis only included SAP and OCT data acquired during baseline visits after initial screening. Of the 90 participants in the trial, 56 had data available for this study. From these 56 participants, 35 (mean age 52.1 ± 11.0) had suitable data supporting all variables studied in our analysis. Reasons for unsuitable data included: OCT image quality was too poor to identify the retinal layers around the fovea; EZ was not continuously apparent throughout the OCT b-scan; evidence of vitreous traction with an epiretinal membrane or cystic spaces; or the SAP reliability factor (RF, the percentage of catch trials that generated either a false positive or false negative response) was greater than 20%. Data was available from both eyes of 25 subjects and one eye from 10 subjects, yielding 60 eyes total.

### Structural data

Spectral-domain OCT imaging with the Heidelberg Spectralis (Heidelberg Engineering, Inc, Heidelberg, Germany) was performed at all VPA trial participating sites with 30° scans designed by protocol to include the maximum foveal depression. Horizontal (H) and vertical (V) line scans were obtained in high-resolution mode with active eye tracking and automatic real-time image averaging, yielding b-scans with approximate resolutions of 3.9 μm axially and 6 μm laterally.

**Fig 1:**
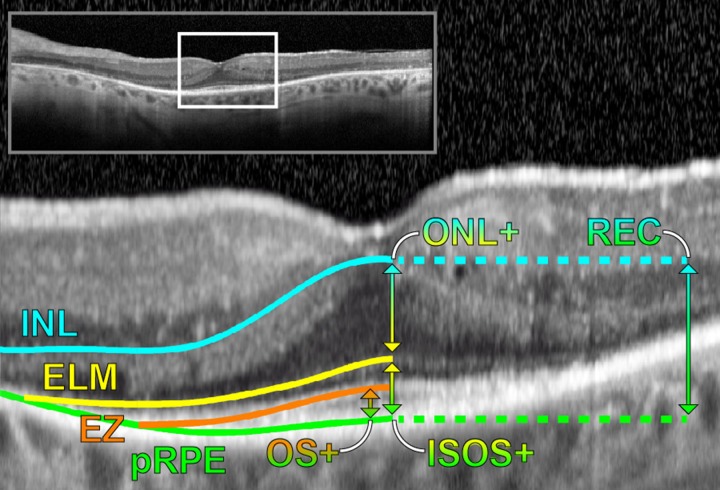
OCT b-scan segmentation. Image from one participant showing the four segmented boundaries and four retinal layers listed in Table 1.

The retinal layers were segmented by a two-step process: automatic segmentation by the HRA/Spectralis (viewing module 6.0.9.0), then manual correction by three trained graders following a segmentation protocol established prior to this study. Graders had no knowledge of the functional test results. As shown in Fig 1, four boundaries were identified: the distal border of the inner nuclear layer (INL), the external limiting membrane (ELM), the photoreceptor inner/outer segment junction or ellipsoid zone (EZ), and the proximal border of the retinal pigment epithelium complex (pRPE). These boundaries generated the four composite layers listed in Table 1. Because the Henle fiber layer appearance is dependent on the scan angle [16], it was included in ONL+ along with the outer plexiform layer to obtain the most reproducible estimate possible of overall photoreceptor integrity. The fovea x and y coordinates were estimated from the point of maximum depression in the H and V b-scans, respectively. The boundary coordinates, the Spectralis infrared (IR) reflectance image of the fundus, and the fovea location were all exported for analysis. All H scans were segmented first, followed by all V scans.

**Table 1:** The outer retinal layers segmented from OCT H and V b-scans.

Layer name	Layer components	Proximal boundary	Distal boundary
ONL+	Outer nuclear layer, Henle fiber layer, outer plexiform layer	INL	ELM
OS+	Photoreceptor outer segment, interdigitation zone	EZ	pRPE
ISOS+	Photoreceptor inner segment, OS+	ELM	pRPE
REC	ONL+, OS+, ISOS+	INL	pRPE

To investigate the effect of the retinal curvature in the b-scan, a flattened OCT data set was generated. Custom software was developed to fit a cubic polynomial to the pRPE boundary and then flatten the OCT images and segmented boundaries by shifting each pixel column vertically by the polynomial model. The flattened data was analyzed in parallel with the original, unflattened scans.

### Functional data

**Fig 2:**
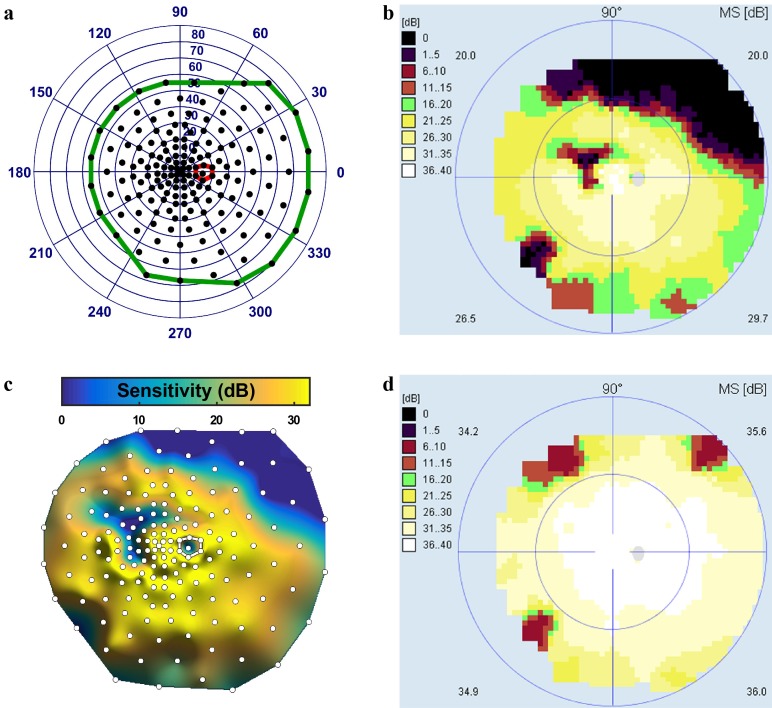
SAP acquisition and HOV representation. (a) Right-eye full-field 164-point SAP test grid pattern, which spans 140° horizontally and 130° vertically. The expected location of the natural blind spot is outlined in red. (b) Example Octopus perimeter sensitivity map for one participant. (c) Top-down view of the interpolated HOV surface for this participant, with the test grid locations shown as white circles. The surface height represents the visual field sensitivity. (d) Octopus sensitivity map for a healthy normal, for comparison.

Full-field automated static visual field testing with an Octopus 900 (Haag-Streit AG, Köniz, Switzerland) was performed at each site using 10 cd/m^2^ background luminance, the GATE-i fast thresholding strategy [17], and a 200 msec size V stimulus. Eyes were tested without dilation with the radially oriented, centrally condensed, binocularly symmetric, 164-point grid shown in Fig 2A. The average test duration was approximately 20 minutes per eye. Duplicate testing was performed and the differential luminance sensitivity (DLS) values from both tests were combined by the weighted arithmetic mean, with weights based on 100 minus the test RF values, to produce a single set of DLS values for the eye. The order of eye testing was right, left, right, left. SAP data (Fig 2B) was resampled onto a uniform grid with 0.36° point spacing using radial basis function interpolation with a thin plate spline kernel, which was validated in a previous study [18]. Resampling produced a three-dimensional sensitivity surface representing the hill of vision (HOV) as shown in Fig 2C.

### Data registration

**Fig 3:**
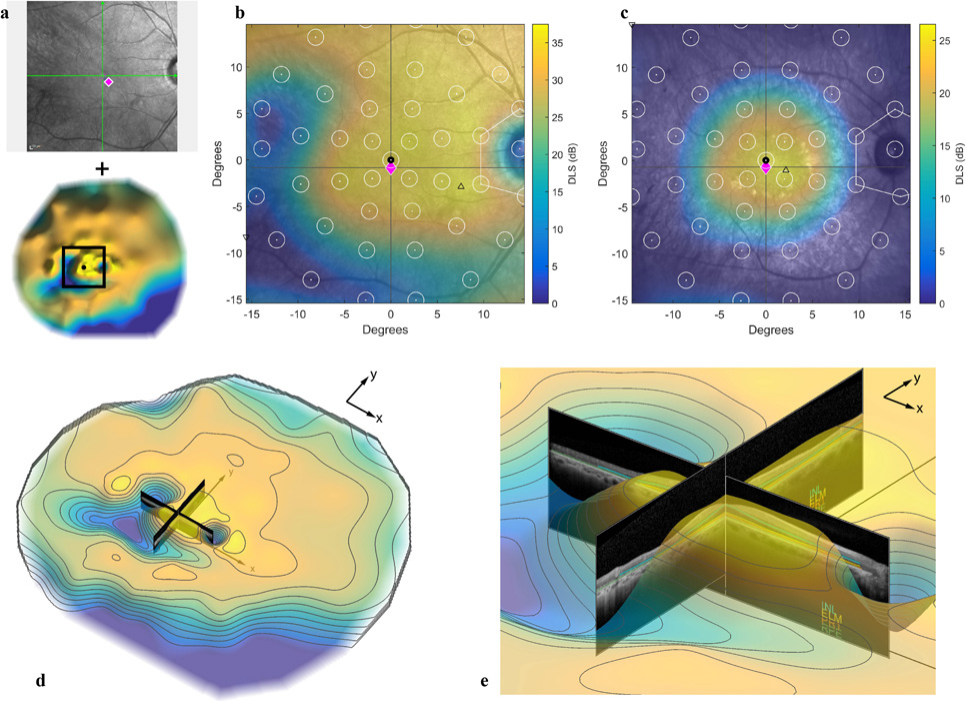
Registration of anatomic and functional data. (a) The IR fundus image (top) and the HOV sensitivity surface (bottom) were first registered automatically by aligning the fovea (magenta diamond) with the center visual field point (black circle). The diamond’s x and y coordinates were derived from the H and V b-scans, respectively. The green lines on the IR image indicate the H and V b-scan locations. The black square on the HOV represents the IR image size (30°). The HOV has been reflected to match the fundus orientation. (b) The HOV was then manually shifted until the natural scotoma was aligned with the optic disc. The white dots indicate the locations of SAP test grid, and the radius of each white circle corresponds to a size V stimulus. (c) For HOVs with no natural scotoma, the optic disc was aligned with the expected location of the blind spot (white polygon). (d) Aligned OCT b-scans and HOV with contour lines for the case shown in b. (e) Close-up of central field. A relationship is evident between steep depressions in the HOV surface and the edges of the EZ (the yellow line labeled PR1 on the b-scans).

Although complementary, SAP and OCT data have disparate information, a low degree of mutuality, and few common features needed by data-driven registration techniques [19]. Alignment is highly dependent on patient fixation—how non-eccentric, stable, and consistent it is between the imaging and functional tests. To overcome this limitation, we developed a semi-automatic registration process using the interpolated HOV sensitivity surface and the 30° IR fundus image, which was automatically aligned with the b-scans by the Spectralis OCT [20] and served as an intermediary between the b-scans and the HOV (Fig 3A). The fovea (x,y) location was marked on the IR image, the visual field (0°,0°) center grid point was aligned with the fovea, and the HOV was superimposed onto the IR image. The resulting overlay (Fig 3B) was inspected to verify alignment between the optic disc and the natural scotoma in the visual field. In some cases there was slight misalignment (<3°), and for these a translational offset was manually added to correct the registration. In cases with no discernible natural scotoma, the optic disc was manually aligned with the expected blind spot location (Fig 3C).

### Biomarker extraction

All biomarkers are listed with their abbreviations in Tables 2 and 3, and were computed separately for each eye with fully automated custom software. For structural biomarkers, the mean thickness and foveal thickness for each of the four layers in Table 1 were extracted from the exported boundary coordinates for the H and V scans. The EZ and ELM widths were each extracted based on the length of the line joining their boundary’s edges.

**Table 2:** Structural biomarkers extracted from OCT b-scans.

Structural biomarker	Biomarker abbreviation
ONL+ thickness	${\text{T}}_{\text{ONL},\text{FOV}}^{\text{H}},\;{\text{T}}_{\text{ONL},\text{AVG}}^{\text{H}}$, ${\text{T}}_{\text{ONL},\text{FOV}}^{\text{V}},\;{\text{T}}_{\text{ONL},\text{AVG}}^{\text{V}}$
ISOS+ thickness	${\text{T}}_{\text{ISOS},\text{MAX}}^{\text{H}},\;{\text{T}}_{\text{ISOS},\text{AVG}}^{\text{H}}$, ${\text{T}}_{\text{ISOS},\text{FOV}}^{\text{V}},\;{\text{T}}_{\text{ISOS},\text{AVG}}^{\text{V}}$
OS+ thickness	${\text{T}}_{\text{OS},\text{FOV}}^{\text{H}},\;{\text{T}}_{\text{OS},\text{AVG}}^{\text{H}}$, ${\text{T}}_{\text{OS},\text{FOV}}^{\text{V}},\;{\text{T}}_{\text{OS},\text{AVG}}^{\text{V}}$
REC thickness	${\text{T}}_{\text{REC},\text{FOV}}^{\text{H}},\;{\text{T}}_{\text{REC},\text{AVG}}^{\text{H}}$, ${\text{T}}_{\text{REC},\text{FOV}}^{\text{V}},\;{\text{T}}_{\text{REC},\text{AVG}}^{\text{V}}$
EZ width	${\text{W}}_{\text{EZ}}^{\text{H}}$, ${\text{W}}_{\text{EZ}}^{\text{V}}$
ELM width	${\text{W}}_{\text{ELM}}^{\text{H}}$, ${\text{W}}_{\text{ELM}}^{\text{V}}$

Abbreviations are for the base: T = thickness, W = width; for the subscript: MAX = maximum, AVG = mean, FOV = foveal; and for the superscript: H = horizontal, V = vertical orientation.

**Table 3:** Functional biomarkers extracted from SAP visual fields.

Functional biomarker	Measurement domain	Biomarker abbreviation
Mean sensitivity, mean defect, loss variance	Global	MS, MD, LV
Total HOV volume & HOV defect volume	Global	S_V_, D_V_
Central 30° HOV volume & HOV defect volume	Global (central HOV)	S_V30_, D_V30_
Maximum HOV sensitivity, HOV defect & associated distances	Global	S_MAX_, D_MAX_, d_S,MAX_, d_D,MAX_
Maximum HOV sensitivity, HOV defect & associated distances	Local (along the b-scan)	${\text{S}}_{\text{MAX}}^{\text{H}},\;{\text{D}}_{\text{MAX}}^{\text{H}},\;{\text{d}}_{\text{S},\text{MAX}}^{\text{H}},\;{\text{d}}_{\text{D},\text{MAX}}^{\text{H}},{\text{S}}_{\text{MAX}}^{\text{V}},\;{\text{D}}_{\text{MAX}}^{\text{V}},\;{\text{d}}_{\text{S},\text{MAX}}^{\text{V}},\;{\text{d}}_{\text{D},\text{MAX}}^{\text{V}}$
HOV slope maximum, HOV slope mean & associated distances	Global	∇S_MAX_, ∇D_MAX_, d_∇S,MAX_, d_∇D,MAX_, ∇S_AVG_, ∇D_AVG_, d_∇S,AVG_, d_∇D,AVG_
HOV slope mean & associated distances	Local (along the b-scan)	$\nabla {\text{S}}_{\text{AVG}}^{\text{H}},\;\nabla {\text{D}}_{\text{AVG}}^{\text{H}},\;{\text{d}}_{\nabla \text{S},\text{AVG}}^{\text{H}},\;{\text{d}}_{\nabla \text{D},\text{AVG}}^{\text{H}},\nabla {\text{S}}_{\text{AVG}}^{\text{V}},\;\nabla {\text{D}}_{\text{AVG}}^{\text{V}},\;{\text{d}}_{\nabla \text{S},\text{AVG}}^{\text{V}},\;{\text{d}}_{\nabla \text{D},\text{AVG}}^{\text{V}}$

Abbreviations are: S = patient’s HOV sensitivity surface, D = HOV defect surface; for the base: d = distance from the center point; for the subscript: MAX = maximum, AVG = mean, V = volume; and for the superscript: H = horizontal, V = vertical orientations for local biomarkers. HOV slope is denoted by ∇.

Functional biomarkers were extracted from the patient’s visual sensitivity data, and were classified as either global (from the entire HOV) or local (along the b-scan). All were computed from both the patient’s HOV and the defect HOV, the difference between an age-adjusted normal HOV and the patient’s HOV. We included standard indices of visual function such as mean sensitivity, mean defect and loss variance [21]. We also extracted HOV characteristics including the maximum sensitivity value and the distance between it and the grid center point. Volumetric biomarkers derived from topographic analysis included the total HOV volume and central 30° volume [18].

**Fig 4:**
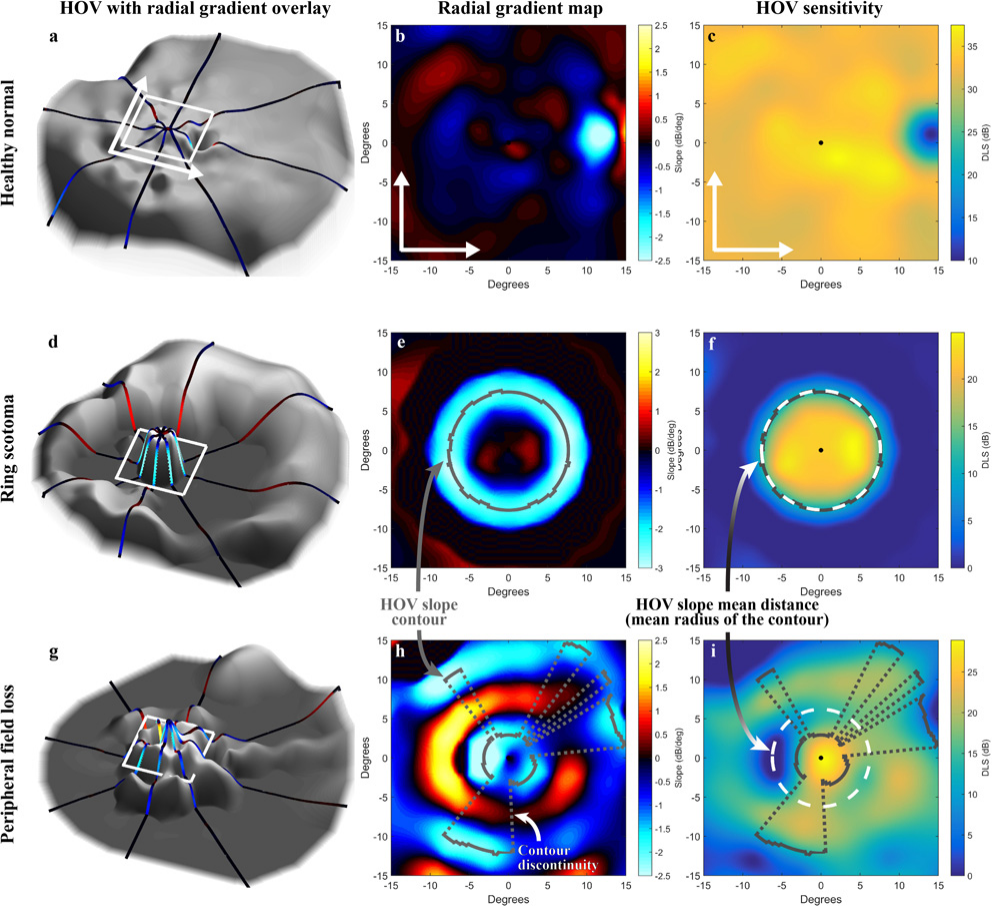
HOV slopes. Each row illustrates a different visual field pattern. (a,d,g) Full-field HOV sensitivity surfaces (gray) overlaid with eight radial spokes color-coded by slope. The white box and arrows indicate the location and orientation of the central 30° region. (b,e,h) The radial gradients of the HOV in the central 30°, with a gray line indicating the HOV slope contour. Discontinuities along the contour are indicated by a dotted gray line. (c,f,i) Top-down view of the HOV sensitivity surfaces. The radius of the white dashed circle is the mean radial distance to the HOV slope contour from the center point (d_∇S,AVG_). The ring scotoma example is from the same data as Fig 3C–3E.

We developed new biomarkers to characterize the topographic slope of the HOV surface and quantify the transition from preserved central field to reduced peripheral field. For convenience, we use the term HOV slope to describe the contour around the center of the HOV where the HOV changes most rapidly. More specifically, the HOV slope contour was defined as the location of the extreme values of the radial gradient, the first derivative taken radially outward along the sensitivity surface. The slope extrema (the most negative slopes for the patient’s HOV, the most positive slopes for the HOV defect) were identified along each of 501 radial spokes emanating from the center point with 0.72° angular separation. For illustration purposes, eight of the 501 spokes are shown in the left column of Fig 4. The locations of the extrema formed a slope contour, which could be discontinuous, on the HOV surface. The following global biomarkers were derived from this contour: the HOV slope maximum value around the contour, the radial distance to this maximum point from the center grid point, the HOV slope mean value around the contour, and the mean radial distance to the contour from the center grid point. Local HOV slope biomarkers were also extracted from just the spokes corresponding to the OCT b-scan orientations.

### Statistical analysis

We analyzed biomarker relationships with mixed-effects modeling (MEM) consisting of a fixed component for the average population trend and a random component for individual deviation from the population mean. MEM controlled for the dependence between biomarkers obtained from eyes of the same person by incorporating inter-eye correlation into the model. All possible pairwise biomarker combinations were analyzed by linear MEM with random intercepts to account for the clustering within participants. Biomarkers from the H and V b-scans were treated separately. For each biomarker pair, we selected the best among the quantitative models in Table 4, which covered a range of relationship trends including linear, nonlinear, and asymptotic behaviors. Age was not a significant covariate as determined by Wald tests and was not included in the models. Model selection was performed by minimizing the Akaike information criterion, which estimates the information loss in a model [22]. For the best model, significance was determined by the F-test p-value after Bonferroni correction [23].

**Table 4:** The three fixed-effects models tested for each structure-function relationship.

Name	Model
Inverse quadratic	Y = a − b/X^2^
Linear	Y = a + bX
Quadratic	Y = a + bX^2^

Here, X is the covariate and Y is the response. For all models, the sign of coefficient b indicates a positive or negative correlation between X and Y.

Model performance was assessed through two coefficients of determination: the marginal coefficient (${\text{R}}_{\text{m}}^{2}$), which measures the proportion of variance explained by the fixed effects only, and the conditional coefficient (${\text{R}}_{\text{c}}^{2}$), which measures the proportion explained by the combined fixed and random effects [24,25]. Both have values between zero and one. A large ${\text{R}}_{\text{m}}^{2}$ means that individuals did not deviate much from the population mean trend line (obtained from the fixed-effects component), and is indicative of a strong association. A large ${\text{R}}_{\text{c}}^{2}$ shows that the full mixed-effects model is valid and the quantitative relationship in the model is trustworthy.

We also assessed differences between right-eye and left-eye biomarkers with paired Wilcoxon signed rank tests, and differences in H and V biomarkers with Wilcoxon rank sum tests. Statistical analyses, custom software development, and data processing and biomarker extraction were all performed in MATLAB 8.5 (MathWorks, Natick, MA, USA). A base level of 0.05 was used for all significance testing.

## Results

### Structure-structure and function-function correlations

Functional measures of the patient’s HOV (for example, S_V_) were tightly coupled to their defect counterparts (D_V_), unsurprising given that they are related through the age-adjusted normal HOV which varies slowly and is nearly constant in the central field [26,27]. There were also other types of strong function-function correlations. Because the global visual field indices MS and MD are intrinsically weighted by the SAP test grid pattern, which was centrally condensed in this case, they were significantly correlated with the central HOV volumes S_V30_ and D_V30_, respectively. The list of all significant function-function and structure-structure associations appears in S1 Table. Because each set of coupled biomarkers shows similar or identical relationship trends, from here forward we primarily focus on unique structure-function relationships. Thus, we present results from the EZ width instead of the ELM width, functional measures derived from the native visual field instead of field defect, and HOV slope mean instead of slope maxima.

### Structure-function relationships

**Fig 5:**
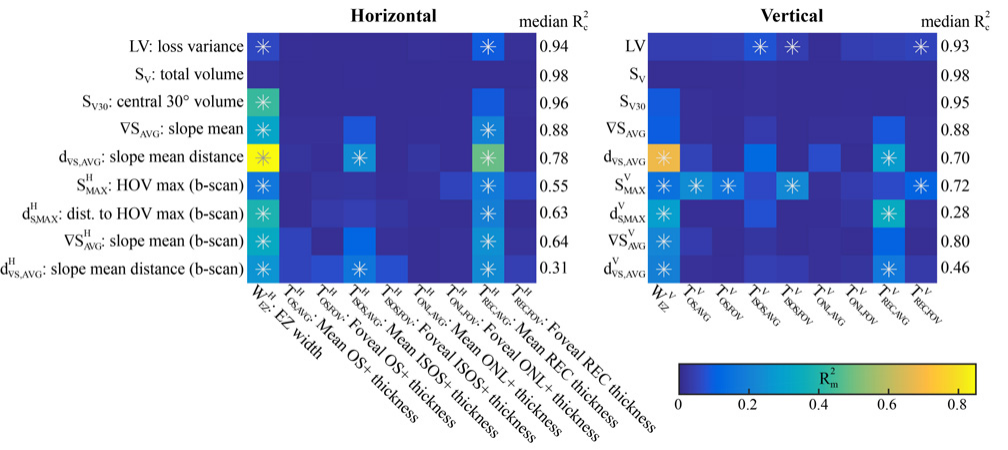
Heat maps of ${\text{R}}_{\text{m}}^{2}$ (fixed-effects goodness of fit) for the best-fit models for each structure-function relationship. Each row is a different functional biomarker, and each column is a different structural biomarker. The first five rows are global measures from the entire HOV, and the last four are local measures from functional data along the b-scan line. For each functional biomarker, the median ${\text{R}}_{\text{c}}^{2}$ (mixed-effects fit) across all structural biomarkers is shown. Superscripts H or V indicate the b-scan direction, and * indicates statistical significance.

Fig 5 summarizes the MEM results. In each model, the covariate X was the structural biomarker and the response Y was the functional one; we found this yielded better model fitting than with structures as responses. For each structure, 9 functional relationships were assessed with a significance level of 0.05/9 = 0.0056 after Bonferroni correction. The MEM fits were generally good to excellent with many yielding ${\text{R}}_{\text{c}}^{2}$ > 0.7, indicating the model captured most of the observed variance. For a given functional biomarker, ${\text{R}}_{\text{c}}^{2}$ values for all structures were fairly consistent; median values are shown in Fig 5. The ${\text{R}}_{\text{c}}^{2}$ values were larger for the five global functional biomarkers than the four local ones due to the increased variability from fewer data points available in local measures. For completeness, the full results including the best-fit models are listed in S2 and S3 Tables.

**Fig 6:**
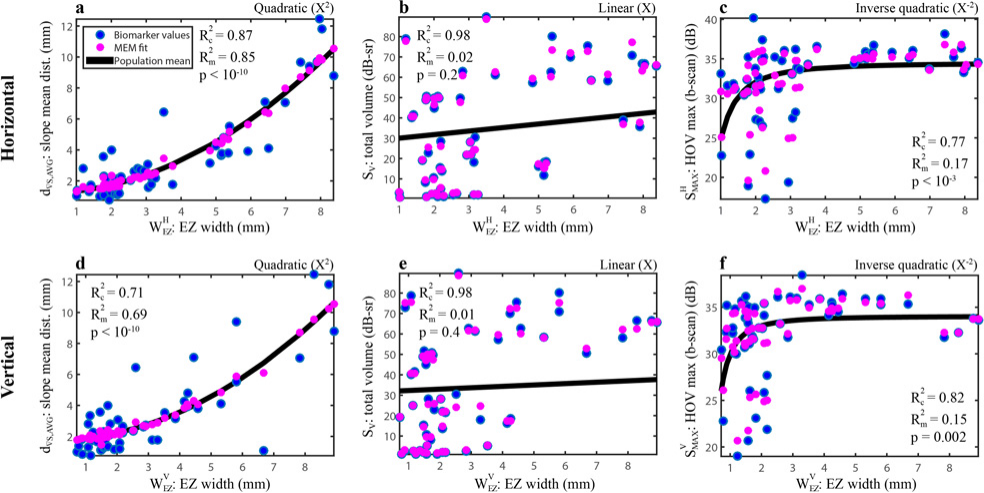
Mixed-effects modeling of several functional relationships with EZ width. The top graphs show the H-oriented measures, and the bottom show the V measures. Raw biomarker values are represented by blue dots and MEM fitted values by magenta dots. The black line is the fixed-effects population mean. These plots depict 6 of the 162 relationships summarized in Fig 5.

For both the H and V orientations, the structure that correlated most frequently and most strongly with the functional measures was the EZ width. It was most strongly associated with the HOV slope mean distance, d_∇S,AVG_; for the horizontal EZ width ${\text{W}}_{\text{EZ}}^{\text{H}}$, this relationship yielded ${\text{R}}_{\text{m}}^{2}$ = 0.85, the largest marginal value observed in this study (Fig 6A). This is compelling because while the EZ width was measured along the midline, d_∇S,AVG_ was computed from the entire visual field. Restricting the functional biomarkers to the local (along the b-scan) HOV slope distances ${\text{d}}_{\nabla \text{S},\text{AVG}}^{\text{H}}$ and ${\text{d}}_{\nabla \text{S},\text{AVG}}^{\text{V}}$, the associations with EZ width were still significant but much weaker with ${\text{R}}_{\text{m}}^{2}$ = 0.26 and ${\text{R}}_{\text{m}}^{2}$ = 0.20, respectively. The EZ widths did not have significant associations with the total HOV volume S_V_ (Fig 6B and 6E) and therefore also not with the mean sensitivity. The EZ width measurements were truncated by the edge of the 30° image in only 6 of the 120 b-scans.

In addition to the EZ width, the mean REC layer thicknesses ${\text{T}}_{\text{REC},\text{AVG}}^{\text{H}}$ and ${\text{T}}_{\text{REC},\text{AVG}}^{\text{V}}$ were also prominent, especially in the H orientation where it was significantly correlated with several functional biomarkers. None of the retinal layer thickness measurements made at the fovea had strong functional correlations, except in the V orientation where they were weakly associated with the loss variance and the local maximum sensitivity ${\text{S}}_{\text{MAX}}^{\text{V}}$.

The distances to the HOV maximum and HOV slope contour generally had stronger associations with structural features than the actual values of the HOV maximum and HOV slope mean. Comparing H and V structure-function relationships, roughly the same number were significant in H and V, the peak ${\text{R}}_{\text{m}}^{2}$ was much larger in H, and the mean ${\text{R}}_{\text{m}}^{2}$ value was slightly larger in H. If a relationship was significant in both H and V, its ${\text{R}}_{\text{m}}^{2}$ tended to be larger in H than in V. These results provide mild support for using H orientations instead of V for future analyses.

### Additional findings

The left-eye and right-eye biomarkers that were statistically different are listed in Table 5. The hybrid structural-functional measures shown in Table 6 were created by combining information from the H and V b-scans. For each of these cases, the H and V biomarkers had similar distributions. The mean HOV defect at the EZ edge, where the IS/OS junction effectively disappears, was 7.6 dB for a size V stimulus. This is comparable to the 8–10 dB defect previously reported when OS thickness effectively goes to zero, albeit with a smaller size III stimulus [7,10]. The HOV sensitivities at the EZ edge were tightly distributed about the mean, yielding a relatively small coefficient of variation of 0.16.

**Table 5:** Biomarkers that were significantly different between eyes (25 right-left pairs).

Biomarkers	Mean difference (right–left eye)	p-value
MS, MD	0.82 dB, –0.82 dB	0.007, 0.007
S_V_, D_V_	1.59 dB-sr, –1.59 dB-sr	0.003, 0.004
S_V30_, D_V30_	0.62 dB-sr, –0.61 dB-sr	0.02, 0.01
${\text{D}}_{\text{MAX}}^{\text{V}}$	–0.43 dB	0.04
${\text{T}}_{\text{OS},\text{AVG}}^{\text{H}},\;{\text{T}}_{\text{OS},\text{AVG}}^{\text{V}}$	–2.89 μm, 1.78 μm	0.007, 0.03
${\text{T}}_{\text{ISOS},\text{AVG}}^{\text{H}}$	–2.31 μm	0.01

**Table 6:** Values of hybrid structural-functional measures.

Measurement	Value (mean ± std)	CV
REC layer thickness at HOV maximum	161 ± 42 μm	0.26
REC layer thickness at HOV defect maximum	71 ± 57 μm	0.80
HOV sensitivity at EZ edge	26.65 ± 4.32 dB	0.16
HOV defect at EZ edge	7.57 ± 4.75 dB	0.63
HOV slope mean at EZ edge	–1.07 ± 0.74 dB/deg	0.69
HOV defect slope mean at EZ edge	0.90 ± 0.73 dB/deg	0.81

Values were computed after combining results from H and V b-scans. CV = coefficient of variation.

All results presented correspond to the unflattened OCT scans. Analysis of the flattened data resulted in no change to the structure-function relationship characteristics or study outcomes. This is consistent with previous work [28] that found flattening had no significant effect.

## Discussion

These findings will be useful for interpreting progression trends and treatment effects in longitudinal studies, and may inform the analysis of natural history studies. Compared to conventional functional endpoints, the functional biomarkers that are strongly correlated with highly repeatable structural biomarkers may be more advantageous. Also, these functional correlates may be appropriate surrogates when the structural biomarkers are unavailable. For example, in patients with very early or advanced disease who have EZ widths that are inconspicuous or extend beyond the OCT field-of-view, HOV slope biomarkers from full-field functional assessments may be suitable replacements.

Many of the relationships described here have not previously been studied. Our preliminary analysis indicated that many relationships would likely be nonlinear, and consequently we chose the three quantitative models in Table 4 because they covered a set of fundamental relationship trends. For superlinear trends, we included the quadratic model instead of an exponential model such as Y = ae^bX^ due to limitations caused by the necessary log transformation of the response variable Y, which would interfere with the model selection [29]. For sublinear and asymptotic trends, we included the inverse quadratic model. All models were linear with respect to the coefficients, and all had the same parsimony with two fixed-effects parameters each. Thus, no model had an intrinsic advantage over the others in terms of data fitting.

Interestingly, many of the right-eye biomarkers for global visual function were larger than their left-eye counterparts. This could be due to ocular dominance—although one previous analysis [30] does not support this hypothesis—or patient fatigue leading to a systematic bias from testing right eyes first. There was no significant difference in visual field RF values between eyes, however there was a significant (p < 0.03) RF worsening over time that suggests a fatigue effect. It is unclear why OS+ layer thicknesses were larger in V for right eyes and larger in H for left eyes. These results indicate that inter-eye differences should be assessed before averaging or otherwise combining data from both eyes.

Some functional measures exhibited increased variance as EZ widths decreased. This can be seen for EZ widths less than 3.5 mm in Fig 6C and 6F, which depict relationships with the maximum sensitivity along the b-scan line. Some patients with similar EZ widths showed maximum sensitivities that differed by 15 dB or more, which would have a considerable impact on vision-related activities [31]. In these patients, functional correlations with the mean ONL+ and REC layer thicknesses were also weak. Further investigation is needed to determine if this larger functional biomarker variance is due to SAP measurement variability, fixation issues, genetic differences, or other causes. A cursory check suggests the larger functional variance in these patients is not due to increased SAP measurement variability. For example, when we analyzed the two SAP exams individually for each subject instead of averaging them, the maximum sensitivity was actually more consistent between the two exams in those subjects having EZ widths smaller than 3.5 mm; the mean absolute inter-exam difference was 0.19 dB versus 0.29 dB for those having EZ widths larger than 3.5 mm. Nonetheless, although test-retest performance was not assessed in this work, other studies have found smaller structural biomarker variance as compared to SAP variability [12,32,33].

One limitation of this study is that, because SAP was performed under photopic conditions, all functional biomarkers described here primarily represent cone function. Thus, comparisons with structural measures like the EZ width, which is supported by both cone and rod photoreceptors [34], are purely observational. Previous structure-function studies [3,6–10,15] made similar comparisons with cone function from light-adapted SAP, and this methodology has practical advantages for retinal degeneration research [35]. Nonetheless, a more rigorous assessment would include subgrouping of patients based on electroretinogram findings to isolate those with diminished rod function or include rod-mediated functional measures from dark-adapted perimetry [36], neither of which were available for this analysis.

Other limitations of this study stem from assumptions about the data. A signal-dependent variance was present in some relationships (for example, Fig 6C and 6F as discussed above), which violates the canonical assumption that response variance is independent of covariate value. Variance stabilization transformation would properly account for this [37,38]. Also, any structure-function analysis such as this will hinge on the multimodal registration accuracy. Because fundus-guided perimetry was not used, we assumed the fovea was the preferred retinal locus (PRL) and the PRL was the (0°,0°) point on the visual field. We also assumed the fovea could be located from the two midline OCT scans. These assumptions may not be true in every case, which is why we manually adjusted the alignment between the HOV and IR fundus image based on the optic disc. When the natural scotoma was visible in the HOV, it correlated well with its expected location (inside the white polygon in Fig 3B, for example); this bodes well for those cases in which the scotoma was not visible and the alignment was based instead on its expected location.

To create each densely sampled HOV surface, we interpolated the raw SAP data. This facilitated the computation of topographic biomarkers such as the HOV volume and slope, since SAP test grid patterns containing thousands of points are impractical. One consequence is that the biomarker values are influenced by the choice of interpolation algorithm. The radial basis function interpolator used here showed good accuracy in a comparison of nonparametric interpolators for static visual fields [39], and the infinite differentiability of thin plate spline interpolation kernel produces HOV surfaces that are smooth and non-faceted [18]. Nonetheless, even though interpolated values were synthesized from measurements, they should not be equated with measurements.

The baseline SAP exams in the VPA trial were tested in duplicate. To reduce variability and improve signal-to-noise ratio, we averaged the threshold measurements from both exams prior to analysis. Averaging even more measurements would improve the reliability of the functional biomarkers, however the trial protocol limited the number of visual field exams to two for time and cost reasons.

For the VPA trial, stimulus size V was chosen for visual field testing based on the expectation that most participants would have moderate to advanced disease. With size V there is the potential for abnormally large threshold sensitivity measurements due to spatial probability summation [40], which could alter the functional biomarkers and interfere with the structure-function relationships. Despite this, size V may be more useful than size III in detecting disease progression and testing patients with more advanced RP [40]. In our analysis, the mean sensitivity and mean defect values across all subjects were 10.7 dB and 19.8 dB, respectively. Even though these values are based on measurements with size V stimuli, they are suggestive of more pronounced visual field loss within the subject group.

## Conclusions

In summary, we have characterized the functional correlates of several structural biomarkers in a cohort of autosomal dominant RP patients. Numerous OCT-SAP structure-function relationships were automatically assessed with mixed-effects analysis and automatic model selection. Nearly every step was performed automatically and reproducibly with custom software; the only exceptions were the OCT segmentation and the SAP-OCT registration, for which we followed pre-established guidelines to improve consistency. We introduced a quantitative measure describing the region of most rapid functional transition, the HOV slope contour mean distance, and we showed that it is very strongly correlated with EZ width and moderately correlated with total photoreceptor layer thickness. Our analysis and modeling framework is extensible and can easily accommodate additional biomarkers and genotype information for future investigations.

## Supporting Information

S1 TableStrongly (${\text{R}}_{\text{m}}^{2}$ > 0.9) and significantly (p < 0.05) correlated structure-structure and function-function biomarker pairs.(DOCX)

S2 TableComplete mixed-effect modeling results for the horizontally (H) orientated biomarkers.Listed for each structure-function relationship are, in order, the best-fit model abbreviation preceded by the correlation direction (+ or -), ${\text{R}}_{\text{c}}^{2},\;{\text{R}}_{\text{m}}^{2}$, and the model p-value. The model abbreviations are X^-2^ = inverse quadratic, X = linear, and X^2^ = quadratic. Significant p-values are in bold.(DOCX)

S3 TableComplete mixed-effect modeling results for the vertically (V) orientated biomarkers.Listed for each structure-function relationship are, in order, the best-fit model abbreviation preceded by the correlation direction (+ or -), ${\text{R}}_{\text{c}}^{2},\;{\text{R}}_{\text{m}}^{2}$, and the model p-value. The model abbreviations are X^-2^ = inverse quadratic, X = linear, and X^2^ = quadratic. Significant p-values are in bold.(DOCX)
