# Supplementary material for: Structure-Function Modeling of Optical Coherence Tomography and Standard Automated Perimetry in the Retina of Patients with Autosomal Dominant Retinitis Pigmentosa
Source: PLoS One. 2016 Feb 4;11(2):e0148022. doi: 10.1371/journal.pone.0148022 (PMC4741516; doi:10.1371/journal.pone.0148022)
Supplement: S1 Table — (DOCX) [file pone.0148022.s001.docx]

| **Biomarker** | **Coupled biomarkers** | **Biomarker** | **Coupled biomarkers** |
| --- | --- | --- | --- |
| $W_{\mathrm{EZ}}^{H}$ | $W_{\mathrm{ELM}}^{H}$ | $d_{\nabla S,MAX}$ | $d_{\nabla D,MAX}$ |
| $W_{\mathrm{ELM}}^{H}$ | $W_{\mathrm{EZ}}^{H}$ | $d_{\nabla D,MAX}$ | $d_{\nabla S,MAX}$ |
| MS | MD, $S_{V}$,$D_{V}$,$S_{V30}$, $D_{V30}$ | ${\nabla S}_{\mathrm{AVG}}$ | ${\nabla D}_{\mathrm{MAX}}$, ${\nabla D}_{\mathrm{AVG}}$, ${\nabla D}_{\mathrm{AVG}}^{H}$, ${\nabla S}_{\mathrm{AVG}}^{V}$, ${\nabla D}_{\mathrm{AVG}}^{V}$ |
| MD | MS, $S_{V}$,$D_{V}$,$S_{V30}$, $D_{V30}$ | ${\nabla D}_{\mathrm{AVG}}$ | ${\nabla S}_{\mathrm{MAX}}$, ${\nabla D}_{\mathrm{MAX}}$, ${\nabla S}_{\mathrm{AVG}}$, ${\nabla D}_{\mathrm{AVG}}^{H}$, ${\nabla S}_{\mathrm{AVG}}^{V}$, ${\nabla D}_{\mathrm{AVG}}^{V}$ |
| $S_{V}$ | MS, MD, $D_{V}$ | $d_{\nabla S,AVG}$ | $d_{\nabla D,AVG}$ |
| $D_{V}$ | MS, MD, $S_{V}$ | $d_{\nabla D,AVG}$ | $d_{\nabla S,AVG}$ |
| $S_{V30}$ | MS, MD, $D_{V30}$ | ${\nabla S}_{\mathrm{AVG}}^{H}$ | ${\nabla S}_{\mathrm{AVG}}$, ${\nabla D}_{\mathrm{AVG}}$, ${\nabla D}_{\mathrm{AVG}}^{H}$ |
| $D_{V30}$ | MS, MD, $S_{V30}$ | ${\nabla D}_{\mathrm{AVG}}^{H}$ | ${\nabla S}_{\mathrm{AVG}}$, ${\nabla D}_{\mathrm{AVG}}$, ${\nabla S}_{\mathrm{AVG}}^{H}$ |
| $S_{\mathrm{MAX}}$ | $S_{\mathrm{MAX}}^{H}$, $S_{\mathrm{MAX}}^{V}$ | ${\nabla S}_{\mathrm{AVG}}^{V}$ | ${\nabla S}_{\mathrm{AVG}}$, ${\nabla D}_{\mathrm{AVG}}$, ${\nabla D}_{\mathrm{AVG}}^{V}$ |
| $S_{\mathrm{MAX}}^{H}$ | $S_{\mathrm{MAX}}$ | ${\nabla D}_{\mathrm{AVG}}^{V}$ | ${\nabla S}_{\mathrm{AVG}}$, ${\nabla D}_{\mathrm{AVG}}$, ${\nabla S}_{\mathrm{AVG}}^{V}$ |
| $S_{\mathrm{MAX}}^{V}$ | $S_{\mathrm{MAX}}$ | $d_{\nabla S,MAX}^{V}$ | $d_{\nabla D,MAX}^{V}$ |
| ${\nabla S}_{\mathrm{MAX}}$ | ${\nabla D}_{\mathrm{MAX}}$, ${\nabla S}_{\mathrm{AVG}}$, ${\nabla D}_{\mathrm{AVG}}$ | $d_{\nabla D,MAX}^{V}$ | $d_{\nabla S,MAX}^{V}$ |
| ${\nabla D}_{\mathrm{MAX}}$ | ${\nabla S}_{\mathrm{MAX}}$, ${\nabla S}_{\mathrm{AVG}}$, ${\nabla D}_{\mathrm{AVG}}$ |  |  |
