# Supplementary material for: Structure-Function Modeling of Optical Coherence Tomography and Standard Automated Perimetry in the Retina of Patients with Autosomal Dominant Retinitis Pigmentosa
Source: PLoS One. 2016 Feb 4;11(2):e0148022. doi: 10.1371/journal.pone.0148022 (PMC4741516; doi:10.1371/journal.pone.0148022)
Supplement: S2 Table — Listed for each structure-function relationship are, in order, the best-fit model abbreviation preceded by the correlation direction (+ or -), Rc2,Rm2, and the model p-value. The model abbreviations are X-2 = inverse quadratic, X = linear, and X2 = quadratic. Significant p-values are in bold. (DOCX) [file pone.0148022.s002.docx]

| H | $\mathbf{W}_{\mathbf{EZ}}^{\mathbf{H}}$ | $\mathbf{T}_{\mathbf{OS,AVG}}^{\mathbf{H}}$ | $\mathbf{T}_{\mathbf{OS,FOV}}^{\mathbf{H}}$ | $\mathbf{T}_{\mathbf{ISOS,AVG}}^{\mathbf{H}}$ | $\mathbf{T}_{\mathbf{ISOS,FOV}}^{\mathbf{H}}$ | $\mathbf{T}_{\mathbf{ONL,AVG}}^{\mathbf{H}}$ | $\mathbf{T}_{\mathbf{ONL,FOV}}^{\mathbf{H}}$ | $\mathbf{T}_{\mathbf{REC,AVG}}^{\mathbf{H}}$ | $\mathbf{T}_{\mathbf{REC,FOV}}^{\mathbf{H}}$ |
| --- | --- | --- | --- | --- | --- | --- | --- | --- | --- |
| **LV** | +X^-2^, 0.97, 0.06, **<10^-3^** | -X^-2^, 0.94, 0.01, 0.3 | -X, 0.94, 0.01, 0.5 | +X, 0.94, 0.01, 0.9 | -X, 0.94, 0.01, 0.7 | +X^-2^, 0.94, 0.02, 0.1 | -X, 0.94, 0.02, 0.2 | +X^-2^, 0.96, 0.09, **0.004** | -X, 0.94, 0.02, 0.2 |
| $\mathbf{S}_{\mathbf{V}}$ | -X, 0.98, 0.02, 0.2 | +X, 0.99, 0.01, 0.3 | -X, 0.98, 0.01, 0.7 | +X, 0.99, 0.01, 0.2 | -X, 0.99, 0.01, 0.4 | +X, 0.98, 0.01, 0.6 | +X^-2^, 0.99, 0.01, 0.3 | -X, 0.98, 0.01, 0.2 | +X^-2^, 0.99, 0.01, 0.3 |
| $\mathbf{S}_{\mathbf{V30}}$ | -X, 0.96, 0.43, **<10^-6^** | -X^-2^, 0.98, 0.01, 0.3 | +X^2^, 0.98, 0.01, 0.4 | +X, 0.98, 0.01, 0.8 | -X, 0.98, 0.01, 0.5 | +X, 0.97, 0.01, 0.8 | +X^-2^, 0.98, 0.02, 0.02 | -X, 0.97, 0.09, 0.009 | +X^-2^, 0.98, 0.02, 0.03 |
| $\boldsymbol{\nabla S}_{\mathbf{AVG}}$ | -X, 0.88, 0.32, **<10^-3^** | -X, 0.91, 0.02, 0.3 | -X^-2^, 0.93, 0.02, 0.2 | +X^-2^, 0.91, 0.08, 0.01 | +X, 0.92, 0.01, 0.7 | +X, 0.92, 0.01, 0.6 | +X^-2^, 0.92, 0.01, 0.3 | +X^2^, 0.88, 0.20, **0.003** | +X^-2^, 0.91, 0.01, 0.4 |
| $\mathbf{d}_{\boldsymbol{\nabla S,AVG}}$ | +X^2^, 0.87, 0.85, **<10^-10^** | +X^2^, 0.81, 0.03, 0.1 | -X^-2^, 0.81, 0.02, 0.4 | +X^2^, 0.79, 0.24, **<10^-3^** | -X, 0.79, 0.01, 0.7 | +X, 0.78, 0.03, 0.2 | -X, 0.80, 0.01, 0.6 | +X^2^, 0.81, 0.49, **<10^-6^** | -X, 0.80, 0.02, 0.5 |
| $\mathbf{S}_{\mathbf{MAX}}^{\mathbf{H}}$ | +X^-2^, 0.77, 0.17, **<10^-3^** | -X, 0.62, 0.01,  1 | -X, 0.60, 0.02, 0.2 | -X, 0.55, 0.03, 0.3 | -X, 0.61, 0.02, 0.6 | +X^-2^, 0.64, 0.02, 0.5 | -X, 0.70, 0.06, 0.06 | -X, 0.67, 0.19, **0.005** | -X, 0.68, 0.06, 0.08 |
| $\mathbf{d}_{\mathbf{S,MAX}}^{\mathbf{H}}$ | -X, 0.68, 0.39, **<10^-4^** | +X, 0.70, 0.01, 0.4 | +X, 0.72, 0.03, 0.1 | -X, 0.63, 0.04, 0.2 | +X, 0.72, 0.03, 0.2 | +X, 0.67, 0.01, 0.6 | +X^-2^, 0.68, 0.01, 0.3 | -X, 0.66, 0.20, **0.004** | +X, 0.68, 0.01,  1 |
| $\boldsymbol{\nabla S}_{\mathbf{AVG}}^{\mathbf{H}}$ | +X^2^, 0.73, 0.34, **<10^-4^** | -X, 0.69, 0.06, 0.07 | -X, 0.66, 0.01, 0.4 | +X^2^, 0.67, 0.12, 0.02 | +X^-2^, 0.64, 0.02, 0.3 | +X, 0.66, 0.01, 0.5 | +X^-2^, 0.66, 0.02, 0.2 | +X^2^, 0.69, 0.25, **<10^-3^** | +X^-2^, 0.65, 0.02, 0.2 |
| $\mathbf{d}_{\boldsymbol{\nabla S,AVG}}^{\mathbf{H}}$ | -X, 0.31, 0.26, **<10^-3^** | -X, 0.43, 0.06, 0.1 | -X, 0.46, 0.07, 0.06 | -X, 0.43, 0.17, **0.005** | -X, 0.44, 0.08, 0.06 | -X, 0.41, 0.01, 0.8 | -X, 0.39, 0.02, 0.3 | -X, 0.42, 0.24, **<10^-3^** | -X, 0.39, 0.04, 0.1 |
