# Supplementary material for: Structure-Function Modeling of Optical Coherence Tomography and Standard Automated Perimetry in the Retina of Patients with Autosomal Dominant Retinitis Pigmentosa
Source: PLoS One. 2016 Feb 4;11(2):e0148022. doi: 10.1371/journal.pone.0148022 (PMC4741516; doi:10.1371/journal.pone.0148022)
Supplement: S3 Table — Listed for each structure-function relationship are, in order, the best-fit model abbreviation preceded by the correlation direction (+ or -), Rc2,Rm2, and the model p-value. The model abbreviations are X-2 = inverse quadratic, X = linear, and X2 = quadratic. Significant p-values are in bold. (DOCX) [file pone.0148022.s003.docx]

| V | $\mathbf{W}_{\mathbf{EZ}}^{\mathbf{V}}$ | $\mathbf{T}_{\mathbf{OS,AVG}}^{\mathbf{V}}$ | $\mathbf{T}_{\mathbf{OS,FOV}}^{\mathbf{V}}$ | $\mathbf{T}_{\mathbf{ISOS,AVG}}^{\mathbf{V}}$ | $\mathbf{T}_{\mathbf{ISOS,FOV}}^{\mathbf{V}}$ | $\mathbf{T}_{\mathbf{ONL,AVG}}^{\mathbf{V}}$ | $\mathbf{T}_{\mathbf{ONL,FOV}}^{\mathbf{V}}$ | $\mathbf{T}_{\mathbf{REC,AVG}}^{\mathbf{V}}$ | $\mathbf{T}_{\mathbf{REC,FOV}}^{\mathbf{V}}$ |
| --- | --- | --- | --- | --- | --- | --- | --- | --- | --- |
| **LV** | +X^-2^, 0.94, 0.04, 0.03 | -X, 0.94, 0.04, 0.02 | +X^-2^, 0.94, 0.05, 0.007 | +X^-2^, 0.95, 0.08, **0.004** | +X^-2^, 0.95, 0.04, **0.003** | -X, 0.93, 0.02, 0.4 | +X^-2^, 0.94, 0.04, 0.05 | +X^-2^, 0.95, 0.05, 0.06 | +X^-2^, 0.95, 0.06, **0.004** |
| $\mathbf{S}_{\mathbf{V}}$ | -X, 0.98, 0.01, 0.4 | -X, 0.98, 0.01, 0.4 | +X, 0.99, 0.01,  1 | -X, 0.99, 0.02, 0.1 | +X, 0.99, 0.01,  1 | -X, 0.99, 0.01, 0.7 | -X, 0.99, 0.01, 0.4 | -X, 0.99, 0.01, 0.9 | -X, 0.99, 0.01, 0.5 |
| $\mathbf{S}_{\mathbf{V30}}$ | +X^2^, 0.95, 0.09, 0.01 | -X, 0.97, 0.02, 0.2 | +X^-2^, 0.97, 0.01, 0.3 | -X, 0.97, 0.02, 0.05 | -X, 0.97, 0.01, 0.6 | -X, 0.97, 0.01, 0.8 | -X, 0.97, 0.01, 0.2 | -X, 0.97, 0.01, 0.3 | +X^2^, 0.97, 0.02, 0.2 |
| $\boldsymbol{\nabla S}_{\mathbf{AVG}}$ | -X, 0.88, 0.10, 0.03 | +X^2^, 0.89, 0.02, 0.2 | -X, 0.90, 0.01, 0.9 | +X^2^, 0.88, 0.04, 0.1 | -X^-2^, 0.91, 0.01, 0.6 | -X^2^, 0.91, 0.02, 0.2 | -X^2^, 0.92, 0.02, 0.2 | +X^2^, 0.90, 0.09, 0.03 | -X^2^, 0.92, 0.02, 0.3 |
| $\mathbf{d}_{\boldsymbol{\nabla S,AVG}}$ | +X^2^, 0.71, 0.69, **<10^-10^** | -X, 0.74, 0.05, 0.1 | -X^2^, 0.80, 0.02, 0.2 | +X^2^, 0.72, 0.13, 0.01 | -X, 0.73, 0.01, 0.5 | +X, 0.70, 0.07, 0.1 | -X, 0.75, 0.01, 0.9 | +X^2^, 0.74, 0.29, **<10^-3^** | +X^-2^, 0.74, 0.02, 0.5 |
| $\mathbf{S}_{\mathbf{MAX}}^{\mathbf{V}}$ | +X^-2^, 0.82, 0.15, **0.002** | +X^-2^, 0.80, 0.24, **<10^-4^** | +X^-2^, 0.81, 0.16, **<10^-3^** | +X^-2^, 0.75, 0.07, 0.07 | +X^-2^, 0.84, 0.23, **<10^-5^** | +X, 0.72, 0.02, 0.4 | -X, 0.78, 0.04, 0.1 | +X^-2^, 0.78, 0.07, 0.09 | +X^-2^, 0.84, 0.11, **0.004** |
| $\mathbf{d}_{\mathbf{S,MAX}}^{\mathbf{V}}$ | +X^2^, 0.31, 0.30, **<10^-4^** | -X, 0.29, 0.01, 0.6 | -X, 0.28, 0.01, 0.7 | -X, 0.28, 0.08, 0.06 | +X, 0.30, 0.01, 0.7 | +X, 0.28, 0.01, 0.4 | +X^-2^, 0.32, 0.03, 0.2 | +X^2^, 0.34, 0.34, **<10^-5^** | +X^-2^, 0.29, 0.02, 0.3 |
| $\boldsymbol{\nabla S}_{\mathbf{AVG}}^{\mathbf{V}}$ | +X^2^, 0.84, 0.22, **0.002** | +X^2^, 0.82, 0.04, 0.1 | -X, 0.84, 0.01,  1 | -X, 0.80, 0.03, 0.2 | +X, 0.85, 0.01, 0.9 | +X, 0.83, 0.02, 0.3 | +X^-2^, 0.84, 0.01, 0.4 | +X^2^, 0.84, 0.11, 0.03 | +X^-2^, 0.84, 0.01, 0.6 |
| $\mathbf{d}_{\boldsymbol{\nabla S,AVG}}^{\mathbf{V}}$ | -X, 0.48, 0.20, **0.003** | -X, 0.48, 0.02, 0.4 | -X, 0.46, 0.02, 0.4 | -X, 0.48, 0.04, 0.1 | -X, 0.47, 0.04, 0.2 | +X, 0.48, 0.02, 0.6 | -X, 0.50, 0.02, 0.3 | -X, 0.53, 0.18, **0.005** | -X, 0.50, 0.04, 0.2 |
